# The T-box transcription factor Brachyury promotes renal interstitial fibrosis by repressing E-cadherin expression

**DOI:** 10.1186/s12964-014-0076-4

**Published:** 2014-11-30

**Authors:** Shiren Sun, Wenjuan Sun, Lin Xia, Limin Liu, Rui Du, Lijie He, Rong Li, Hanmin Wang, Chen Huang

**Affiliations:** Department of Nephrology, Xijing Hospital, Fourth Military Medical University, 169 Chang le West Road, Xi’an, Shaanxi Province 710032 China; State Key Laboratory of Cancer Biology, Fourth Military Medical University, Xi’an, Shaanxi China

**Keywords:** TGF-β1, Brachyury, E-cadherin, Renal fibrosis

## Abstract

**Background:**

Epithelial-to-mesenchymal transition (EMT) induced by TGF-β1 is one of well-recognized factors contributing to renal fibrosis. However, the underlying molecular mechanisms of EMT are not fully understood. Brachyury, an evolutionarily conserved transcription factor, was recently identified as an important factor promoting EMT in human carcinoma cell lines. There is no evidence that Brachyury is involved in renal tubular EMT.

**Results:**

Our results demonstrated that Brachyury was prominently induced in TGF-β1-treated human proximal tubular epithelial (HK-2) cells and that this induction was accompanied by changes characteristic of EMT. Blockage of Brachyury expression by short interfering RNA (siRNA) in HK-2 cells effectively reversed the TGF-β1-induced EMT phenotype. Brachyury induction repressed E-cadherin transcription; the E-cadherin promoter contains a Brachyury binding site, and decreased expression of E-cadherin occurred in Brachyury-overexpressing cells when they were transfected with reporter constructs using the promoter. This effect was partially mediated by Slug and Snail, as knockdown of Snail and Slug by siRNA effectively reversed Brachyury-mediated EMT and partially restored E–cadherin expression. The expression of Brachyury also presented in a rat model of obstructive nephropathy and in tubulointerstitial fibrosis tissues of IgA nephropathy, suggesting that it may have a role in EMT and renal fibrosis *in vivo*.

**Conclusion:**

Our results demonstrate for the first time that Brachyury plays an important role in regulating TGF-β1–mediated renal EMT and could be an attractive target for progression of renal disease therapies.

## Introduction

Renal interstitial fibrosis is characterized by accumulation of extracellular matrix (ECM), which is a result of increasing myofibroblasts [1]. The origins of interstitial myofibroblasts include activation of resident fibroblasts or pericytes, expansion of perivascular fibroblasts, infiltration of circulating bone marrow-derived fibrocytes and epithelial to mesenchymal transition (EMT) and/or endothelial-mesenchymal (EndoMT) transition [2]. Tubular EMT is a well-characterized process that renal tubular cells lose their epithelial phenotype and transform to a mesenchymal phenotype, and it has been proved to promote the generation of interstitial myofibroblasts and finally leads to renal fibrosis [3]. Although the role of EMT in renal fibrosis is debated in several studies, EMT is increasingly recognized as one of the major pathways in the disease [3-5].

EMT is regulated by numerous factors in different ways, and transforming growth factor β1 (TGF-β1) is considered as the chief one [3]. TGF-β1 is an important well-established regulator of EMT which regulates the transdifferentiation of tubular epithelial cells into myofibroblasts in renal fibrosis [6-12]. Emerging data indicate that TGF-β1 induced EMT via Smad-dependent and Smad-independent pathways [13,14]. In the Smad-dependent signaling pathway, TGF-β signals are transduced by transmembrane serine/threonine kinase type II and type I receptors. The activated type I receptor kinase phosphorylates Smad2 and Smad3, which then form a complex with Smad4 that translocate to the nucleus and regulates gene expression negatively or positively [15]. In renal fibrosis, it was also demonstrated that activation of Smad2/3 was involved in TGF-β1 induced EMT [16-18]. Although it is widely accepted that TGF-β1 plays an important role in promoting tubular EMT, the mechanism by which TGF-β1 induces tubular EMT remains largely unknown.

Brachyury, an evolutionarily conserved and T-box transcription factor [19-21], is vital for embryonic development in all vertebrates [22-31]. Recent studies have shown that Brachyury promotes EMT involved in cancer progression and metastasis by repression of E-cadherin transcription, leading to a loss of E-cadherin-mediated cell–cell adhesion; thus, activation of EMT regulators might play important roles in mediating the invasion, migration, and metastatic activity of different carcinoma cells [32,33]. Furthermore, Latinkic BV et al found that induction of a homolog of Brachyury in *Xenopus* is regulated by TGF-β signals [34]. Based on these results, we hypothesized that Brachyury might contribute to TGF-β1-induced renal tubular EMT. However, no studies have evaluated the possible role of Brachyury in renal tubular EMT.

In this study, we characterized the effect of Brachyury on TGF-β1-induced tubular EMT and investigated the underlying mechanisms. *In vitro* and *in vivo* studies revealed that Brachyury is functionally involved in promoting tubular EMT by repressing E-cadherin transcription. Our study suggests that TGF-β1-induced Brachyury expression might contribute to the pathogenesis of progressive renal fibrosis.

## Results

### Brachyury is induced rapidly during TGF-β1-mediated EMT

**Figure 1:**
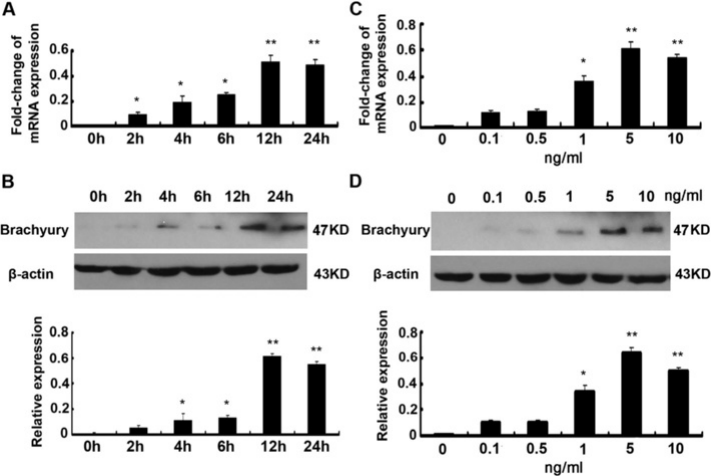
**Brachyury is induced rapidly during TGF-β1–mediated EMT. (A)** qRT-PCR analysis of Brachyury of HK2 cells that were incubated with 5 ng/ml TGF-β1 for various periods. The results shown were representative of three independent experiments. The histogram showed the average volume density corrected for the loading control, glyceraldehyde-3-phosphate dehydrogenase (GAPDH) (n = 3). ^*^ *P* < 0.05 and ^**^ *P* < 0.01 compared with the control*.* **(B)** Western blotting analyses demonstrate that TGF-β1 induced Brachyury protein expression in a time-dependent manner. A representative blot from three independent experiments was shown. The histogram showed the average volume density corrected for the loading control, β-actin(n = 3). ^*^ *P* < 0.05 and ^**^ *P* < 0.01 compared with control*.* **(C)** qRT-PCR analysis of Brachyury of HK2 cells that were incubated at various concentrations of TGF-β1 for 24 h as indicated. The results shown were representative of three independent experiments. The histogram showed the average volume density corrected for the loading control, GAPDH (n = 3). ^*^ *P* < 0.05 and ^**^ *P* < 0.01 compared with control*.* **(D)** Western blotting analyses demonstrate that TGF-β1 induced brachyury protein expression at various concentrations of TGF-β1 for 24 h. A representative blot from three independent experiments was shown. The histogram showed the average volume density corrected for the loading control, β-actin (n = 3). ^*^ *P* < 0.05 and ^**^ *P* < 0.01 compared with control*.*

As determined by qRT-PCR, Brachyury was induced at the mRNA level in TGF-β1-treated HK-2 cells. Figure 1A shows that the level of Brachyury mRNA was increased at 2 h and that the increase was sustained at least until 24 h. Western blotting revealed that Brachyury protein was abundant 4 h after TGF-β1 treatment, and the increase was sustained for at least 24 h (Figure 1B). Brachyury mRNA was induced after treatment of tubular epithelial cells with 1 ng/ml TGF-β1, and the maximal induction was observed at 5 ng/ml TGF-β1, as demonstrated by qRT-PCR (Figure 1C). The dose-response curve of Brachyury protein expression in cells treated with TGF-β1 is shown in Figure 1D. Peak expression was observed at 5 ng/ml TGF-β1 as well. Brachyury expression in HK-2 cells was not increased by a further increase in the TGF-β1 concentration.

### Brachyury mediates TGF-β1-induced EMT in HK-2 Cells

**Figure 2:**
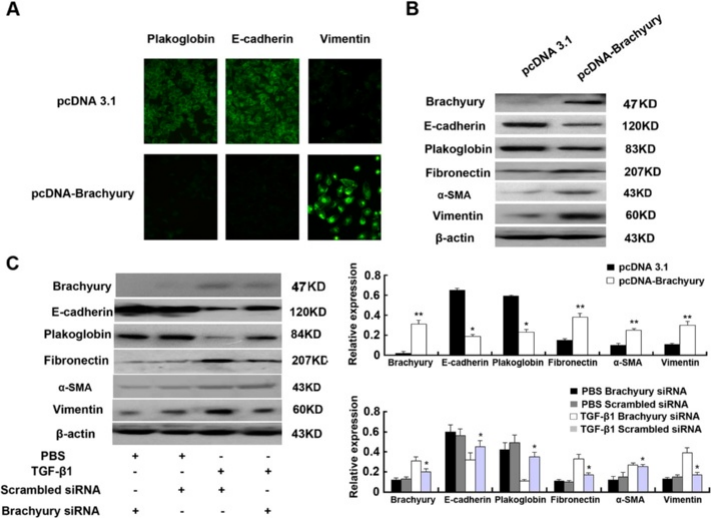
**Brachyury mediates TGF-β1-induced EMT in HK-2 cells. (A)** Immunofluorescence analysis of plakoglobin, E-cadherin and vimentin expression in pcDNA3.1 control and pcDNA3.1 Brachyury transfected HK2 cells. The green signal represents the staining of the corresponding protein. **(B)** Western blot analysis of plakoglobin, E-cadherin, fibronectin, α-SMA and vimentin expression in pcDNA3.1 control and pcDNA3.1 Brachyury transfected HK2 cells. A representative blot from three independent experiments is shown (Top). The histogram shows the average volume density normalized to the loading control, β-actin (Bottom) (n = 3). ^*^ *P* < 0.05 and ^**^ *P* < 0.01 compared with the pcDNA3.1 empty vector transfected cells. **(C)** Western blots analysis of plakoglobin, E-cadherin, fibronectin, α-SMA, vimentin and Brachyury expression in Brachyury-specific siRNA or scrambled siRNA transfected HK2 cells. A representative blot from three independent experiments is shown (Left). The histogram shows the average volume density normalized to the loading control, β-actin (Right) (n = 3). ^*^ *P* < 0.05 and ^**^ *P* < 0.01 compared with the parental cells and control cells.

We next investigated whether Brachyury is required for TGF-β1-induced EMT. We upregulated Brachyury expression by transfecting cells with pcDNA3.1-Brachyury plasmid. Figure 2A shows immunofluorescent staining of tubular epithelial cells. Compared with controls cells that were transfected with pcDNA3.1 control plasmid, overexpression of Brachyury resulted in decreased staining of E-cadherin and plakoglobin, while vimentin staining was dramatically increased. Western blotting analysis was used to evaluate changes in protein expression in these cells. As illustrated in Figure 2B, after transfection of cells with the pcDNA3.1-Brachyury vector, Brachyury expression was increased substantially compared to empty vector controls. It is interesting that overexpression of Brachyury suppressed expression of the epithelial cell markers, E-cadherin and plakoglobin, in tubular epithelial cells, whereas the levels of fibronectin, α-smooth-muscle actin (α-SMA) and vimentin were increased. Our results show that Brachyury could cause EMT of tubular cells *in vitro.*

To further examine the potential role of Brachyury in TGF-β1–mediated EMT of tubular epithelial cells, transfection with Brachyury small interfering RNAs (siRNAs) or scrambled siRNAs was performed in HK-2 cells in the absence or presence of TGF-β1. As shown in Figure 2C, transfection of HK-2 cells with Brachyury-specific siRNA resulted in substantial inhibition of Brachyury protein expression in HK-2 cells with TGF-β1 treatment. Compare with the control cells, the expression of epithelial and mesenchymal markers changed in TGF-β1–treated HK-2 cells after transient transfection with siRNA-Brachyury. As expected, knockdown of Brachyury partially increased E-cadherin and plakoglobin expression and prevented the overproduction of fibronectin, α-SMA and vimentin in response to TGF-β1 stimulation (Figure 2C *lanes 3 versus lanes 4*). This result may reflect the existence of other downstream effectors of TGF-β1 signaling that promote changes in EMT characteristics. In any case, Brachyury seems to be required to mediate TGF-β1–initiated E-cadherin suppression in tubular epithelial cells and at least partly mediates TGF-β1-induced EMT phenotypes *in vitro*.

### The effect of Brachyury was mediated by Snail and Slug

**Figure 3:**
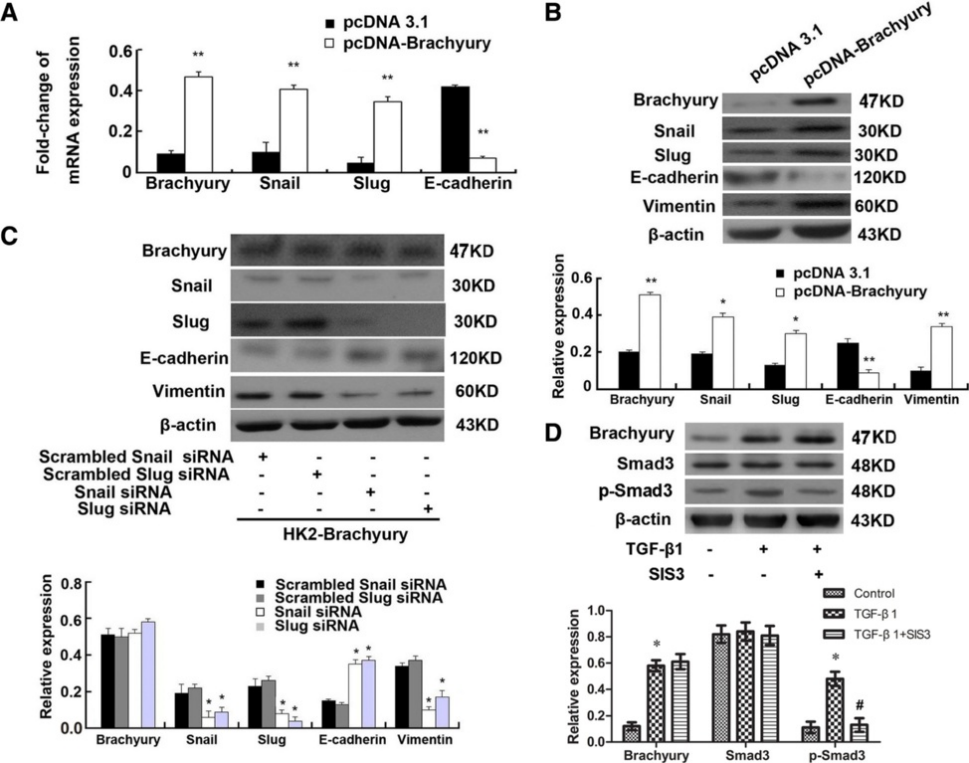
**The effect of Brachyury was mediated by Snail and Slug. (A)** qRT-PCR was used for analysis of Brachyury, E-cadherin, Slug and Snail mRNA levels in HK-2-Brachyruy cells. The results shown were representative of three independent experiments. The histogram showed the average volume density corrected for the loading control, GAPDH (n = 3). ^*^ *P* < 0.05 and ^**^ *P* < 0.01 compared with the pCDNA3.1 empty plasmid transfected cells*.* **(B)** Snail, Slug, E-cadherin, vimentin protein level were detected by Western blotting 24 h after transfection with pcDNA3.1-Brachyury or pcDNA3.1 control in HK2 cells. A representative blot from three independent experiments is shown (Top). The histogram shows the average volume density normalized to the β-actin (Bottom) (n = 3). ^*^ *P* < 0.05 and ^**^ *P* < 0.01 compared with the pCDNA3.1 empty plasmid transfected cells*.* **(C)** Expression of Brachyury, Snail, Slug, E-cadherin and vimentin was analyzed by western blotting in Snail-specific siRNA, Slug-specific siRNA or scrambled siRNAs as indicated. A representative blot from three independent experiments is shown (Top). The histogram shows the average volume density normalized to the β-actin (Bottom) (n = 3). ^*^ *P* < 0.05 and ^**^ *P* < 0.01 compared with the scrambled siRNAs transfected cells*.* **(D)** Western blots analysis of Brachyury, Smad3 and p-Smad3 protein levels in HK2 cells pretreated with TGF-β1 or TGF-β1 and SIS3 (Top). The histogram shows the average volume density normalized to the β-actin (Bottom) (n = 3). ^*^ *P* < 0.05 compared with the untreated control cells. ^*#*^ *P* < 0.05 compared with the TGF-β1 treated cells.

Reduction of E-cadherin expression is recognized as a common event during the course of EMT [35]. Snail and Slug, two members of the zinc finger transcription factor family, have been previously shown to efficiently suppress E-cadherin expression during the course of EMT [36-38]. Fernando RI and his colleagues confirmed that Brachyury promotes EMT by repression of E-cadherin transcription in carcinoma cells, partially via zinc finger transcription factor [32]. To determine whether Snail and Slug are involved in the effect of Brachyury in HK-2 cells, the pcDNA3.1 Brachyury plasmid and the pcDNA3.1 plasmid were transfected into HK-2 cells respectively, and Snail and Slug mRNA levels were measured by qRT-PCR 24 h after transfection. As shown in Figure 3A, some expression of Snail and very little expression of Slug mRNA were found in control HK-2 cells, while the expression of both was enhanced in HK-2-Brachyury cells. Next, we elucidated the simultaneous changes of these transcription factors protein levels in Brachyury-overexpressing HK-2 cells. As indicated in Figure 3B, control HK-2 cells showed some expression of Snail and Slug, while the expression of both proteins was driven in the Brachyury-overexpressing cells. To further clarify whether Snail and Slug are involved in the effect of Brachyury in renal epithelial cells, we silenced the expression of Snail and Slug in Brachyury-overexpressing cells respectively. The protein expression in these cells was detected by western blotting after 24 h transfection. Figure 3C showed that E-cadherin expression was dramatically increased in Brachyury-overexpressing cells that had been transfected with siRNAs for transcription factors (Brachyury, Snail, Slug) respectively, whereas the level of vimentin was decreased. These results illustrate that Brachyury affects E-cadherin expression at least partially through mediation by Slug and Snail in HK-2 cells. Smad3 was an important signal transducer which could be activated by TGF-β1. To explore whether smad3 was involved in TGF-β1-induction of Brachyury, SIS3, an inhibitor of Smad3 phosphorylation was used. As shown in Figure 3D, TGF-β1 significantly induced the expression of Brachyury and the phosphorylation of smad3. Smad3 inhibitor treatment significantly inhibited the phosphorylation of smad3 induced by TGF-β1, but did not affect the expression level of Brachyury. This data indicated that TGF-β1 induced the expression of Brachyury in a Smad3-independent manner.

### Confirmation of the presence of a Brachyury-binding site in the E-cadherin promoter

**Figure 4:**
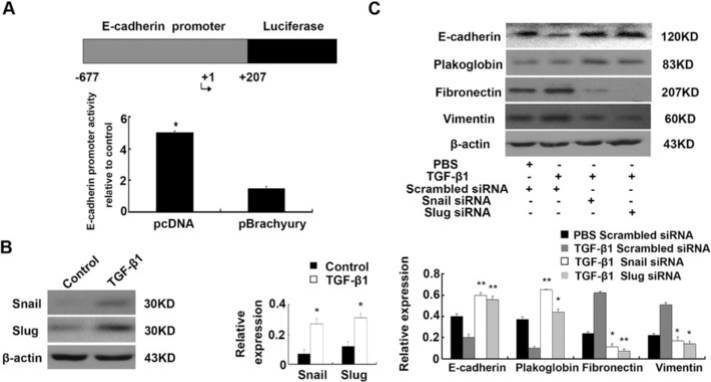
**Confirmation of Brachyury-binding site in E-cadherin promoter and Snail siRNA and Slug siRNA prevents TGF-β1-induced EMT. (A)** Relative reporter activity of the E-cadherin promoter report gene compared with the control cell line. The mean values from three independent experiments were shown. ^*^ *P* < 0.05 compared with the control. **(B)** Western blots analysis of Snail and Slug in HK2 cells that were incubated with 5 ng/ml TGF-β1 for 24 h. A representative blot from three independent experiments is shown (Left). The histogram shows the average volume density normalized to the β-actin (Right) (n = 3). ^*^ *P* < 0.05 and ^**^ *P* < 0.01compared with the control. **(C)** Western blots analysis of E-cadherin, plakoglobin, fibronectin and vimentin expression in Snail–specific siRNA, Slug–specific siRNA or scrambled siRNAs transfected HK2 cells. A representative blot from three independent experiments is shown (Top). The histogram shows the average volume density normalized to the loading control, β-actin (Bottom). (n = 3). ^*^ *P* < 0.05 and ^**^ *P* < 0.01 compared with the scrambled siRNAs transfected cells and TGF-β1 treated cells*.*

Because E-cadherin appeared to be regulated by Brachyury, we further studied whether Brachyury targets E-cadherin. When we examined the E-cadherin promoter for putative Brachyury-binding sites (BBSs), only one site that shares homology with the Brachyury-binding half-site consensus sequence TCACACCT was found. To detect transcriptional regulation of E-cadherin by Brachyury, a luciferase reporter was used. A reporter construct containing the *luciferase* gene under the control of an 884-bp fragment of the human E-cadherin promoter (position –677 to +207) were constructed. As shown in Figure 4A, after HK-2-pBrachyury cells were transiently transfected with it, the reporter activity was repressed compared with the control cells, and the reporter activity decreased by 6.5 ± 1.82-fold. These findings indicated that E-cadherin transcription maybe directly or indirectly regulated by Brachyury in HK-2 cells and suggested that Brachyury binding to the E-cadherin promoter might suppress E-cadherin expression.

### Knockdown of Snail and Slug expression prevents TGF-β1-induced EMT

To investigate whether the Brachyury-Snail/Slug signaling pathway is required for TGF-β1 mediated EMT in tubular epithelial cells, we identify the expression of Snail and Slug in TGF-β1-treated HK-2 cells as well. As demonstrated in Figure 4B, both expression of them were up-regulated. Next, HK-2 cells were transiently transfected with siRNA-Snail, siRNA-Slug or scrambled siRNAs. As shown in Figure 4C, transfection with Snail-specific or Slug-specific siRNA resulted in increased E-cadherin and plakoglobin expression and reduced levels of fibronectin, α-SMA and vimentin compared with the controls. These results indicate that TGF-β1 mediates the mesenchymal transition of HK-2 cells through the Brachyury-Snail/Slug signaling pathway.

### Expression of Brachyury is induced in the fibrotic kidney

**Figure 5:**
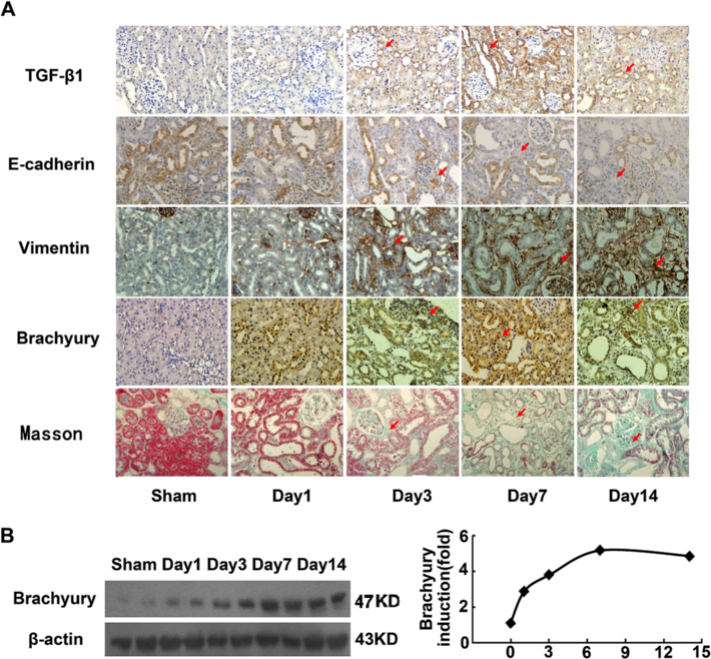
**Brachyury expression is induced in the fibrotic kidney. (A)** Immunohistochemical analysis for TGF-β1, Brachyury, vimentin and E-cadherin in the kidney tissue of UUO rats and sham-operated rats. The tubule interstitial fibrosis was analyzed using a Masson’s trichrome staining kit. Original magnification × 400. **(B)** Western blot analyses revealed the induction of Brachyury protein in the fibrotic kidney. Samples from two representative animals were used at each time point. A representative blot from three independent experiments is shown (left). Relative Brachyury protein levels (fold induction over the sham control) after correction with actin are presented (right).

To address the relevance of Brachyury induction to renal fibrosis *in vivo*, we examined the expression of Brachyury in the evolution of renal interstitial fibrosis induced by unilateral ureteral obstruction and renal biopsies. As shown in Figure 5A, immunohistochemical staining showed that Brachyury and TGF-β1 were mainly expressed with high level at 7d after UUO, whereas it was almost no staining in sham-operated kidneys. The tubule interstitial fibrosis was increased in a time-dependent manner as shown by Masson’s trichrome staining assay. The staining of E-cadherin was decreased, and vimentin staining was increased in the kidneys of these animals in a time-dependent manner. The level of Brachyury protein began to increase 3d after surgery, and maximal induction was observed at 7d, as determined by Western blot analyses (Figure 5B).

**Figure 6:**
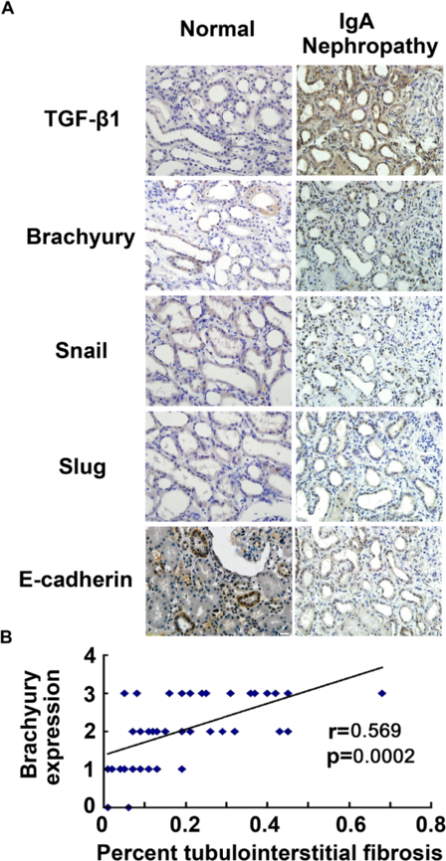
**Brachyury is induced specifically in the degenerated tubular epithelia of the fibrotic kidney. (A)** Immunohistochemical analysis shows inverse correlation of TGF-β1, Brachyury, Snail, Slug and E-cadherin in IgA Nephropathy and normal renal biopsy samples from patients. TGF-β1,Brachyury, Slug, Snail-negative/E-cadherin preserved case. TGF-β1,Brachyury,Slug, Snail-positve/E-cadherin reduced case. Original magnification × 400. **(B)** Correlation plots of tubulointerstitial expression of active Brachyury and tubulointerstitial fibrosis. Scatter plot with fitted value intervals for percent tubular expression of Brachyury and tubulointerstitial fibrosis.

After demonstrating that Brachyury is activated in a rat model of renal fibrosis, we next examined the extent of Brachyury in renal biopsy tissues from IgA nephropathy that had different degrees of tubulointerstitial injury. IgA nephropathy accounts for 30-50% of the progression to end-stage renal disease (ESRD) within 20 years of clinical follow-up [39,40], and therefore represents a major portion of progressive kidney diseases. As shown in Figure 6A, strong staining of Brachyury/Snail/Slug was observed in the nuclei of renal tubular epithelial cells and reduced expression of E-cadherin in IgA nephropathy patients with tubulointerstitial fibrosis, and the positive rate of three transcription factors was 74.4% (32/43), 48.8% (21/43) and 55.8% (24/43) respectively. While little Brachyury was induced in tubular epithelial cells and high level of E-cadherin in normal kidney. The collection with tubulointerstitial fibrosis and Brachyury expression was analysed by linear analysis (p = 0.0002, r = 0.569 Figure 6B). Furthermore, the association with the IHC grading of Snail/Slug and that of Brachyury was significant (P = 0.021, P = 0.031; Table 1). An absent or reduced expression of E-cadherin was observed in 68.8% (33/48) from IgA nephropathy patients and expression of E-cadherin was inversely associated with Brachyruy (p = 0.047; Table 1). These results confirm that Brachyury overexpression occurs during tubular EMT and renal interstitial fibrogenesis *in vivo*.

**Table 1:** **Correlation of the IHC grading of Brachyury, E-cadherin, Slug and Snail in 43 IgA nephropathy patients**

			**Brachyury IHC results**				**p-Value** ^*^
			**Negative**		**Positive**		
			**Grade 0**	**Grade 1+**	**Grade 2+**	**Grade 3+**	
E-cadherin	Preserved		2	6	7	5	0.047
	Loss or reduced		0	3	9	11	
Snail IHC results	Negative	Grade 0	0	5	3	2	0.021
		Grade 1+	2	2	5	3	
	Positive	Grade 2+	0	2	6	7	
		Grade 3+	0	0	2	4	
Slug IHC results	Negative	Grade 0	0	5	2	1	0.031
		Grade 1+	1	2	5	3	
	Positive	Grade 2+	1	2	3	8	
		Grade 3+	0	0	6	4	

^*^Estimated by χ2 test.

## Discussion

EMT is observed in both animal models and human renal biopsy tissues of renal fibrosis with variable degrees and has been increasingly recognized as one of the major pathways which lead to renal fibrosis [41,42]. Although the importance of EMT in renal fibrosis is controversial, there are some evidences for better understanding of the issue. Inoue *et al*. demonstrated that the emergence of EMT-derived fibroblasts arises in a disease-specific and strain-dependent manner [43]. Thus, the different experimental conditions including animal models, mouse strain and the type of genetic alteration used should be taken into consideration when studying the EMT involvement in renal fibrosis. The TGF-β superfamily proteins are well recognized as a primary controller of the pathogenesis of renal fibrosis [44]. Many molecules in this family, especially TGF-β1, have been identified as positive regulators of this process because of their ability to facilitate the EMT program [45]. However, the underlying molecular mechanisms are still not fully understood. In this study, we show through *in vitro* and *in vivo* experiments that Brachyury is involved in regulating TGF-β1–mediated EMT in renal tubular cells and present evidence for a role of Brachyury in TGF-β1-induced renal tubular EMT.

A group of transcription factors known as EMT transcription factors can drive the process of EMT; these transcription factors include Snail, Slug, Twist, and the recently identified T-box family member Brachyury [32,33,37,46,47]. Brachyury, an evolutionarily conserved transcription factor, is indispensable for the formation of posterior mesoderm and axial development in all vertebrates [19,20]. Mutation of *Xbra*, the *Xenopus* homolog of Brachyury, results in failure of differentiation of early embryonic cells into mesodermal tissues [48]. To our knowledge, members of the T-box transcription family, including Brachyury, preferentially bind to the E-cadherin promoter [49]. E-cadherin, which is crucial for the formation of stable adherent junctions, plays a vital role in epitheliogenesis during embryonic development and in maintaining the state of mature epithelial differentiation [50,51]. Downregulation of E-cadherin is recognized as one of the most common occurrences in early stage EMT. A recent study reported that Brachyury and repressed E-cadherin transcription might be involved in EMT in carcinoma cells [32]. Latinkic BV et al found that Brachyury expression was greatly enhanced by TGF-β during the development of the embryo [34]. It was also demonstrated that TGF-β1 induced Brachyury expression in human prostate and lung cancer cell lines. TGF-β1-mediated Brachyury upregulation in human prostate and lung cancer cell lines was relevant to tumor invasiveness, chemotherapy and EMT [52]. However, no studies have suggested that Brachyury is regulated by TGF-β1 in renal tubular tissue. Here, we found that Brachyury is involved in mediating tubular EMT induced by TGF-β1 *in vitro* and in a rat model of obstructive nephropathy. This result was in accordance with the study of Larocca C. and colleagues [52]. However, the induction of Brachyury occurs over different time courses in the two systems; the maximal expression of Brachyury protein was observed 4 h after TGF-β1 treatment *in vitro* but 3d after UUO *in vivo*. Brachyury protein expression was sustained for a longer period of time *in vivo*, demonstrating that Brachyury induction has a prolonged functional role in tubular epithelium. The reason for the difference in time course between Brachyury protein induction *in vitro* and *in vivo* is still unclear, but it is likely that other factors are involved in the stability of Brachyury protein *in vivo*. In addition, TGF-β1 action was mimicked by overexpression of Brachyury, resulting in loss of E-cadherin and plakoglobin, and the expression levels of vimentin and fibronectin were upregulated. Knockdown of Brachyury expression increased epithelial cell marker expression in tubular epithelial cells; moreover, the levels of fibronectin and vimentin were suppressed. The fact that Brachyury knockdown partly reversed TGF-β1-induced EMT may indicate that other downstream effectors of TGF-β1 signaling exert to EMT. On the basis of our results, we can confirm that Brachyury is associated with the induction of EMT in tubular epithelial cells and that it at least partly mediates the TGF-β1-induced EMT phenotype *in vitro*.

It has been shown that the Brachyury half-consensus binding sequence in the E-cadherin promoter is at position –645 (TCACACCT). In this work, we used the luciferase reporter system to confirm that Brachyury is able to bind to the E-cadherin promoter and found that the efficiency is significant. Muller CW et al and Rodriguez M et al also found binding of T-box proteins to a half consensus site of the E-cadherin promoter [49,53]. Activated E-cadherin transcriptional repressors play specific physiological roles during development, but they can re-emerge and become activated in disease. Snail, zinc finger transcription factors, repress E-cadherin expression by its sequence-specific DNA binding abilities and competes with basic helix-loop-helix (bHLH) proteins for binding to the E-box [37,38]. A pathogenic role of Snail in tubulointerstitial injury is well established [54]. ZEB factors and E12/E47 have been confirmed as promoters of EMT and metastasis, Snail and Slug (them all implicated in tumor development) firmly have the roles in promoting EMT in tubular cells, in the fibrotic kidney and in human tumor cells [54-56], and Brachyury in human tumor cells [32].This observation leads us to speculate that Snail, Slug and Brachyury expression are correlated with EMT in HK-2 cells. In this work, we showed that overexpression of Brachyury induces a concerted upregulation of two different E-cadherin transcriptional repressors, Snail and Slug, at the mRNA and protein level in tubular cells and that E-cadherin expression is silenced, whereas knockdown of Snail or Slug by siRNA effectively elicits changes characteristic of EMT and partially restores E-cadherin expression. Slug and Snail each also seem to play a partial role in mediating TGF-β1-induced EMT. These results are in agreement with previous studies that report Brachyury transcriptional repression of E-cadherin via zinc finger transcription factors [49,53] and other studies in which it was reported that decreased expression of Slug or Snail is associated with the restoration of E-cadherin expression in a variety of cancers [57-59]. In addition, it was observed that removal of Snail also induced a decrease of Slug in Brachyury overexpressing cells, and removal of Slug decreased Snail expression, too (Figure 3C). So, it was difficult to clarify whether the effects were mediated by Snail or Slug or both proteins based on our data. No literature documented the interaction of Slug and Snail until now. The underlying mechanism needs further study. Because Brachyury, Slug and Snail all participate in the repression of E-cadherin expression, it appears that multiple pathways can lead to EMT. However, in HK-2 cells, Brachyury, partially mediated by Slug and Snail, clearly plays a leading role in EMT. In this new signaling pathway involved in TGF-β1 mediated tubular EMT provided in the present study, Brachyury is a new potential target for renal fibrosis treatment.

Our investigation of the role of Brachyury in IgA nephropathy of renal tissue showed that Brachyury was expressed at a high level in tubulointerstitial fibrosis tissues of IgA nephropathy, while little Brachyury was induced in tubular epithelial cells of normal kidney. Reduced expression of E-cadherin was also observed under conditions of high expression of Brachyury in tubular epithelial cells. We also observed that Brachuyry expression in the tubules has strong association with tubulointerstitial fibrosis. These observations suggest that activated Brachyury might participate in a common pathway that results in dysregulation of the E-cadherin expression pattern in tubular EMT, and this may have relevance to renal interstitial fibrosis *in vivo*.

The findings reported here show that Brachyury may be a functional factor that has a vital role in TGF-β1–mediated tubular EMT and renal interstitial fibrosis. Further analysis is needed to characterize the role of Brachyury in the progression of renal disease in more detail and to determine whether Brachyury is an attractive target for renal disease therapy.

## Materials and methods

### Cell culture and experimental conditions

The human proximal tubular epithelial cell line (HK-2) was cultured in Dulbecco’s modified Eagle’s medium/F12 medium (Invitrogen Inc.) supplemented with 10% fetal calf serum (FCS) (Gibco-BRL Life Technologies, Burlington, Ontario, Canada). The cells were seeded at 1.5 × 10^6^ cells/100 mm-diameter dish for 3-4 days at 37°C in the presence of 10 ml of medium in a humidified atmosphere of 5% CO_2_. All experiments were carried out with confluent cultures. Serum-starved HK-2 cells were treated with TGF-β1 (R & D Systems, Minneapolis, MN) at a concentration of 5 ng/ml for various periods of time and for 24 h with various dose-responses as indicated. SIS3 (Santa Cruz Biotechnology, Santa Cruz, CA), an inhibitor of Smad3 phosphorylation was introduced to the medium to elucidate the pathway involved in TGF-β-induction of Brachyury. The cells were then collected for qRT-PCR and western blotting analysis.

### Kidney biopsies

Renal biopsy samples diagnosed at the Xijing hospital from 2010 to 2011 were reviewed, and cases with sufficient material (at least ten glomeruli) were selected for further study after informed consent was obtained, according to the guidelines of the Xijing hospital ethics committees (Permit Number: XJYYLL10042). Renal biopsy samples were processed by standard techniques for immunochemistry; clinical diagnosis was made on the basis of the immunochemical straining. The IgA nephropathy was obtained from the standard pathology report.

### Animal preparation

Male Sprague–Dawley rats weighing 160–180 g were obtained from the laboratory animal center of our university (Fourth Military Medical University, Xi’an, China). All animals underwent unilateral ureteral obstruction (UUO) under diethyl ether anesthesia. Left ureteral ligation was performed at the level of the lower pole of the kidney. After surgery, the animals were returned to their cages and given free access to food and water. Sham-operated animals underwent the same surgical intervention except for ureter ligation. Rats were sacrificed at various time points after surgery, and their kidneys were immediately excised; some were fixed with 4% paraformaldehyde, and others were snap-frozen in liquid nitrogen until analysis. This study was performed in strict accordance with the recommendations in the Guide for the Care and Use of Laboratory Animals of the National Institutes of Health. The protocol was approved by the Committee on the Ethics of Animal Experiments of the Fourth Military Medical University (Permit Number: 11098).

### Protein preparation and western blotting

Kidney tissues (0.1 mg) and cells (2 × 106) were extracted with lysis buffer (50 mmol/L Tris-HCl pH 8.0, 150 mmol/L NaCl, 0.1% SDS, 1% Nonidet P-40, 0.5% sodium deoxycholate, 0.02% sodium azide, 100 μg/ml PMSF, 1 μg/ml aprotinin). The tissue samples were then homogenized by ultrasonic vibration and heated at 95°C for 5 minutes. Cell lysates were centrifuged at 4°C for 5 minutes at 10000 rpm, and the protein-containing supernatant was removed to fresh tubes. Protein concentration was determined using the Bradford method.

For western blotting, total proteins (80 μg) were electrophoresed on 10% SDS polyacrylamide gels and then transferred to nitrocellulose membranes (Millipore, Bedford, MA). After blocking with 10% fat-free milk in TBS (20 mmol/L Tris, 0.15 mol/L NaCl (pH 7.0), 0.1% Tween 20), the membranes were incubated at 4°C overnight with a primary antibody: anti-Brachyury (Sigma Chemical Co.) diluted 1:1000; anti-Snail (Sigma Chemical Co.) diluted 1:200; anti-Slug (Sigma Chemical Co.) diluted 1:1000; anti-E-cadherin (Santa Cruz Biotechnology) diluted 1:200; anti-fibronectin (Sigma Chemical Co.) diluted 1:1000; anti-α-smooth muscle actin (α-SMA) (Abcam) diluted 1:500; anti-Smad3 (Santa Cruz Biotechnology) diluted 1:800; anti-p-Smad3 (Santa Cruz Biotechnology) diluted 1:800; anti-plakoglobin (Santa Cruz Biotechnology) diluted 1:200; or anti-vimentin (Santa Cruz Biotechnology) diluted 1:200. After repeated washing, the membranes were incubated with horseradish-peroxidase-conjugated anti-rabbit or anti-mouse secondary antibody (Santa Cruz Biotechnology) diluted 1:2000. The bands were visualized using the enhanced chemiluminescence system (Amersham Pharmacia Biotech) and exposed to Kodak X-OMAT film (Rochester, New York, USA). Western blotting for β-actin was performed as an internal control for sample loading using mouse monoclonal antibody (1:3000, Sigma Chemical Co.). Autoradiograms were quantified by densitometry (software: Bio Image IQ). Relative protein levels were calculated by reference to the amount of β-actin protein.

### RNA extraction and quantitative RT-PCR analysis

Total RNA was extracted from HK-2 cells using Trizol Reagent (Invitrogen; Carlsbad, CA) and then reverse-transcribed (RT) (cDNA Synthesis Kit, Bio-Rad, Hercules, CA). The RT products were amplified using a TaqMan Gene Expression Assays kit (Applied Biosystems). All PCRs were done in triplicate. The sequences of the primers were as follows: Brachyury, forward 5’-GAC GCA AAA GAA CGT TCT GAC-3’ and reverse 5’- AGG ACT GCG TGG TGA TAC AG-3; Snail, forward 5’-ACT CGG TAC CAG TCT ACT ATC-3’ and reverse 5’-TGG CGC GAA TTT TTA CCC TTC-3’; Slug, forward 5’- AAT AGG ATT TCC CAT AGG AAG AGA -3’ and reverse 5’- AGT TCA ACA ATG GCA ACC AG -3’; for glyceraldehyde-3-phosphate-dehydrogenase (GAPDH), forward 5’-GGC AAA TTC AAC GGC ACA GTC-3’ and reverse 5’-GCT GAC AAT CTT GAG TGA GTT-3’. The protocol comprised 45 cycles of 95°C for 5 sec, 60°C for 30 sec, and 72°C for 1 min each. Reactions were run on a real-time PCR system (ABI PRISMH 7700, Applied Biosystems). GAPDH was chosen as the reference gene in the present study. A △CT value was calculated for each sample by subtracting the threshold cycles (CT) of the GAPDH from the CT value of the detected gene. All samples in each group were normalized to the △CT value of a control sample (△△CT). The relative expression was calculated using the expression 2^-△△CT^.

### Immunohistochemistry and immunocytochemistry

For immunohistochemistry, sections 2-mm thick tissue slides were made. Slides were dewaxed, rehydrated, incubated with 3% hydrogen peroxide for 30 min, and blocked in 10% normal goat serum for 1 h. The slides were then incubated with primary antibodies including anti-Brachyury (1:1000; Sigma Chemical Co.), anti-Snail (1:2000; Sigma Chemical Co.), anti-Slug (1:1000; Sigma Chemical Co.), anti-E-cadherin (1:100; Santa Cruz Biotechnology), and anti-vimentin (1:200; Santa Cruz Biotechnology) at 4°C overnight. The sections were incubated with biotinylated goat anti-rabbit or anti-mouse IgG as the secondary antibody, and the antibody reactions were visualized using diaminobenzidine (DAKO, Tokyo, Japan). Non-immune goat IgG or rabbit IgG were used as negative controls. The slides were counterstained with hematoxylin, dehydrated and mounted. A 0–3 relative scale was used to grade the amount of Brachyury/Snail/Slug immunostaining: 0, 5% staining; 1+, 5–25% staining; 2+, 25–50% immunostaining; 3+, .50% immunostaining. A negative/positive relative scale was used to grade the amount of E-cadherin immunostaining: negative (0), loss or reduced membrane expression of E-cadherin; positive (1), preserved membrane expression of E-cadherin in tubular epithelial cells.

For immunocytochemical analysis, HK-2 cells were cultured on sterile glass coverslips in 24-well plates. The coverslips were fixed with 4% paraformaldehyde for 15 min at room temperature. The coverslips were washed with phosphate-buffered saline (PBS) and permeabilized for 5 min with 0.1% Triton X-100 in PBS. After blocking with 10% normal goat serum for 1 h, the coverslips were incubated with primary antibodies including anti-vimentin (1:100; Santa Cruz Biotechnology), anti-E-cadherin (1:100; Santa Cruz Biotechnology), and anti-plakoglobin (1:100; Santa Cruz Biotechnology). The slides were incubated with FITC-conjugated goat anti-mouse or anti-rabbit IgG secondary antibodies at room temperature for 1 h and analyzed by confocal laser scanning microscopy.

### Masson’s trichrome staining assay

The 2-mm thick tissue slides were stained with Masson’s trichrome using a Masson kit (Fuzhou Maixin Biotechnology LTD, Fuzhou, China) according to the manufacturer’s instruction.

### Plasmid and transfection

Full-length human Brachyury cDNA was cloned into the pcDNA3.1 plasmid (GenScript); the pcDNA3.1 empty plasmid was used as a control. For silencing, siRNA-Brachyury (sc-29820, Santa Cruz Biotechnology Inc.), siRNA-Snail (sc-38398-SH, Santa Cruz Biotechnology Inc.), and siRNA-Slug (sc-38393, Santa Cruz Biotechnology Inc.) were used. Scrambled siRNAs contained the same nucleotide content as the selected siRNAs but in a random sequence, and the scrambled siRNAs had no calculated target gene specificity as assessed by BLASTing against all human sequence databases. Cells were plated and grown to 70–90% confluency without antibiotics. Transfections were performed with Lipofectamine 2000 (Invitrogen AB., Lidingo, Sweden), according to the manufacturer’s protocol.

### Bioinformatics analysis of the Brachyury binding site

A genomic region of -1000 bp upstream of the E-cadherin transcriptional initiation site was determined using the NCBI Genomic BLAST program. This DNA sequence was then pasted into DNA Strider 1.0 software, which was used to locate a putative Brachyury-binding site (BBS). The search was based on compilations of functional BBS and the Brachyury-binding half-site consensus sequence TCACACCT.

### E-cadherin promoter assay

HK-2 cells stably transfected with pcDNA3.1 or pBrachyury were cultured in a 24-well plate (50,000 cells per well). Clear 24-well plate were maintained in parallel to assess confluence. After 24 hours, cells were transfected with 200 ng E-cadherin promoter or control luciferase vector (Promega, WI, USA). The luciferase activity of the cultures was measured and quantitated in a luminometer using the Dual-Luciferase Reporter Assay System (Promega) after 48 h transfection. Results were expressed as the mean of the ratio between the firefly luciferase activity and the renilla luciferase activity.

### Statistical analysis

Each experiment was repeated at least three times. Bands from Western blot or RT-PCR were quantified by Quantity One software (Bio-Rad). Relative protein and mRNA levels were calculated by referring them to the amount of β-actin or GAPDH respectively. Data were analyzed by standard statistical methods, including linear regression correlation test, ANOVA, the t-test and χ2 test using SPSS (version 14.0). Numerical data are presented as mean ± SD. Significance was assessed at P < 0.05.

## Abbreviations

EMT: Epithelial-to-mesenchymal transition
TGF-β1: Transforming growth factor β1
FCS: Fetal calf serum
GAPDH: Glyceraldehyde-3-phosphate-dehydrogenase
siRNAs: interfering RNAs
α-SMA: α-smooth-muscle actin
